# Everyday Life and Social Contacts of Dementia and Non-Dementia Residents over 80 Years in Long-Term Inpatient Care: A Multi-Level Analysis on the Effect of Staffing

**DOI:** 10.3390/ijerph182111300

**Published:** 2021-10-27

**Authors:** Melanie Zirves, Ibrahim Demirer, Holger Pfaff

**Affiliations:** 1 Graduate School GROW—Gerontological Research on Well-Being, Faculty of Human Sciences and Faculty of Medicine, University of Cologne, 50931 Cologne, Germany; 2 Institute of Medical Sociology, Health Services Research, and Rehabilitation Science (IMVR), Faculty of Human Sciences and Faculty of Medicine, University of Cologne and University Hospital Cologne, 50933 Cologne, Germany; ibrahim.demirer@uk-koeln.de (I.D.); holger.pfaff@uk-koeln.de (H.P.)

**Keywords:** long-term care, nursing home, nurse staffing, multi-level analysis, aged 80 and over

## Abstract

The relationship between nurse staffing, physical outcomes of residents, as well as quality of care receives major attention. The impact of staffing levels on residents’ ability to organize their everyday life and maintain social contacts, however, has not been analyzed to date. This study examines whether a relationship between the staff-to-resident ratio for registered nurses and nursing home residents with and without dementia aged over 80 exists. Secondary data collected in the project inQS (indikatorengestützte Qualitätsförderung) were used (*n* = 1782, mean age = 88.14). The analyzed cross-sectional data were collected in winter 2019 in facilities of the Diocesan Caritas Association in Germany. A sum score formed from variables measuring residents’ abilities to independently organize their everyday life and maintain social contacts functioned as the dependent variable. A multi-level regression analysis was performed. The results revealed that the ability of residents without dementia was significantly associated with the staff-to-resident ratio of registered nurses. This was not true for residents with dementia. For the latter, however, whether the facility offers a segregated care unit turned out to be significant. Additional and longitudinal research is indispensable to explain the inequality between the two groups analyzed.

## 1. Introduction

Currently, the age group of people older than 80 years of age is the fastest-growing in the world [1]. The proportion of the population over 80 years of age as well as the population suffering from dementia at this age is also rising rapidly in Germany [2,3]: 6.8% of the German total population were aged 80 or older in 2018 [4]. By 2050, this group will represent 13% [5]. The proportion of people suffering from dementia will increase from 1.59 million in 2020 to 2.35 million in 2050 [6]. Since people suffering from dementia have a high care dependency [7], this growing proportion is expected to be accompanied by an increase in institutional care in the next years [2,8,9,10,11].

Being able to organize everyday life and social contacts independently is part of the self-determination as well as the control experienced, and therefore also has an impact on the well-being and health of people in need of care [12,13,14]. In the group of people over 80, the ability to organize one’s own everyday life and to maintain social contacts may be physically and/or cognitively restricted. Care facilities or the staff working there can actively promote these abilities by activating the residents and supporting them [12], which is why McCabe et al. [14] argue that nurses have a key role in fostering the social relationships that residents choose and thereby improving quality of life.

Residents with cognitive impairments presumably need more support than those without impairment, which is why, depending on abilities or type of illness, residents need adjusted care. Therefore, different skills on the staff side are needed [15]. By 2035, however, a shortage of 307,000 in nursing staff in Germany is expected [16]. This shortage raises the question of how to adequately care for the growing number of heterogeneous nursing home residents [17].

As autonomy and the maintenance of social relationships empower residents’ quality of life [14], the growing number of oldest residents makes an investigation of social, organizational, and nursing aspects of good quality of life in nursing homes necessary [18]. The relationship between physical outcomes, quality of life outcomes of residents, as well as organizational determinants—such as the quantity and quality of staff—has been repeatedly and widely documented in research studies [3,7,19,20,21,22,23,24]. These studies, however, mainly used quality measures such as pressure ulcers, falls, lengths of hospital stay, medication errors, patient or resident satisfaction, depression, anxiety, and quality of life [2,9,15,20,25,26]. Everyday life and engagement in everyday activities are associated with habits of action and make it possible to orient and occupy oneself [27]. By maintaining social contacts—both inside and outside the facility—relationships can be cultivated and social exchanges remain possible [14,27]. McCabe et al. [14] studied, among other things, the contribution of the relationship between staff and residents to the promotion of residents’ quality of life. In their study, Mondaca et al. [28] address the limited impact that older and frail nursing home residents have on everyday activities. The role of staff is also highlighted, but not in terms of how they influence everyday activities and social contacts of the residents. Although residents over 80 years of age are over-represented in nursing homes [10] and this age group should receive more attention in research due to its rapid growth, no study could be identified that explicitly examined factors influencing everyday life routines and social contacts of residents over 80 years of age. Additionally, no study was found that simultaneously addressed the effect of staffing. In order to bridge this gap, we analyzed the question: To what extent do nursing staff levels (as an organizational or structural feature of facilities) influence those residents’ maintenance of everyday life and social contacts?

This question is highly relevant, especially in times of the COVID-19 pandemic [26]. Residents in inpatient long-term care particularly suffer from restricted contact with the outside world. Their (social) life is confined to a very limited space [29]. In the course of this, the relationship with the staff takes on increasing importance. As fewer relatives are allowed to visit nursing homes, staff furthermore have a new role in activating residents and facilitating social contact for older people: be it within the nursing home or—through technical support such as telephones and video telephony, i.e., using temi robots—to the outside world [13,29,30,31].

### Conceptual Framework and State of Research

According to Donabedian’s paradigm [32], structural quality increases the likelihood of process quality and outcome quality. However, it cannot guarantee them. According to Donabedian, structural quality includes material resources (e.g., equipment, financial resources), human resources (e.g., number and qualification of staff), and organizational characteristics (e.g., organizational structure of staff, compensation methods) [32,33]. Rothgang et al. [34] identified facility staffing—both qualitatively and quantitatively—as the most important structural aspect in long-term care. McCloskey et al. [17] see staffing as “the most objective, reliable, and measurable form of data available [that] can be used as a starting point to understand the conditions within nursing homes”. Whether a facility is adequately staffed may affect (other) structure, process, and outcome determinants of quality of care [26]. The conceptual model of the present study therefore assumes that staffing levels are related to residents’ outcomes, resulting from the assumption that a high staff-to-resident ratio and a high ratio of professional staff are necessary for caregivers to adequately perform their tasks [9,21,35,36].

On 1 January 2017, a new definition of the need for care was introduced in Germany [37]. The focus was placed on independence and abilities, i.e., on competencies and not on deficits of people in need of care. The associated demand for better quality of care can only be guaranteed with appropriate staffing levels and only if the required personnel can be recruited as well as trained adequately [22,34]. Thus, the German government adopted a comprehensive package of measures on 3 June 2019. It includes strategies to recruit more personnel, to guarantee better working conditions and better pay, and to increase the attractiveness of the nursing profession [34,38]. Nevertheless, one of the most urgent challenges facing German society in the 21st century is to ensure an adequate number of nursing staff in long-term care [34].

Staffing statistics in Germany require at least 50% registered nursing staff [34]. However, this percentage is not always met in practice [3,21]. In addition, no requirements are defined for the other qualification levels. Konetzka et al. [15] concluded that taken together, most studies showed that more registered nurses “in absolute sense and as a proportion of total hours” contribute to better residential outcomes. Kim et al. [39] reported inconsistent findings concerning the impact of registered nurses (RN) on quality nursing home care. However, the authors highlighted that the current RN staffing standard should also be reviewed for effectiveness. While Greg et al. [40], Castle et al. [41], and Backhaus et al. [23] showed that a continuous relationship between staff-mix and quality of care does not necessarily exist, different longitudinal studies have shown positive relationships concerning staff-mix, skill-mix, pressure ulcers, and urinary tract infections [15,21,22]. Harrington et al. [22] argued that the strongest relationships exist between RN and residential outcomes. In their review, Griffiths et al. [25] concluded that higher levels of nursing assistants (NA) contribute to negative outcomes of hospital patients concerning falls, pressure ulcers, and satisfaction, while they provided strong evidence for a positive relationship between a skill-mix richer in RN and patient outcomes. Castle and Anderson [21] claimed that it is the sum of all caregivers that influences quality of care and quality of life for residents. Therefore, not only RN staff should be targeted [21], although the authors did not provide any statements on how the qualification levels should be depicted. Furthermore, Rothgang et al. [34] criticized that it is not specified how the qualification levels of staff should be distributed below the required 50% of RN in German long-term care facilities.

We did not identify a study directly analyzing the impact of staff on the maintenance of everyday life and social contacts. However, Lowndes et al. [8] identified that staff themselves have little to no time for engaging socially with residents and that staff cannot focus enough on residents’ social needs in Canada, Germany, and Norway. Especially during evening shifts that often are understaffed, it seems impossible to enable residents to be socially active [8]. Lowndes et al. [8] highlight that residents suffering from dementia have less visitors and are less socially connected than people without dementia. Many of the needs of residents with dementia for social care, community involvement, company, and daytime activities are not met [8,42]. Harmer and Orrell [43] blamed low staffing levels, as well as staff attitudes of prioritizing physical over social needs and routines within facilities that limit autonomy, for the lack of opportunities for residents with dementia to participate in activities they experience as meaningful. Based on their literature review, Lowndes et al. [8] called for studies that examine the physical environment and its impact on residents’ (with and without dementia) participation in everyday life.

McCabe et al. [14] examined the relationship between staff and residents and the role of staff in promoting autonomy and choice. According to Ryan and Deci’s self-determination theory [44], autonomy and choice, along with feelings of competence as well as social and emotional connectedness to others, play a significant role in residents’ well-being and quality of life. This also holds true for residents suffering from dementia [45]. Vaismoradi et al. [30] showed that residents’ perception that staff and family recognize their need for autonomy is associated with increased vitality, greater well-being, and lower mortality in nursing homes. Since most residents rate choice and control about everyday life as very important, but are unsatisfied with the amount of choice and control available, healthcare professionals should minimize the loss of control that nursing home residents often experience when moving to a long-term care facility [13]. In line with this, Abbott et al. [31] suggested that staff should have time to listen to the wishes of nursing home residents regarding the maintenance of their social contacts. Haugan et al. [2] claimed that nursing home staff nurses are “the most important providers of social reinforcement”. Furthermore, in terms of, for example, the theory of selection, optimization, and compensation (SOC), staff have a role to play in enabling residents to socialize and manage everyday life by opening up new channels (e.g., writing emails) when others have closed down (e.g., hearing sufficiently on the phone) [31]. By additionally creating an interpersonal relationship [7], staff can positively influence the environment in which social contacts can be maintained [14]. One nursing approach that aims to preserve self-determination, respects “the individualized rhythms of daily life” [13], and thus focuses on the individual with his or her autonomy and social needs, is person-centered care [30,42,46]. However, this approach is rarely used.

Based on the noted aspects, the intention of the present study was to answer the central question mentioned in the introduction while analyzing for the following hypotheses:

**Hypothesis** **1** **(H1).** *The higher the staff-to-resident ratio of registered nurses in a facility, the more likely a facility is able to ensure that its residents over 80 years of age have the capacities to manage everyday life and social contacts independently*.

**Hypothesis** **2** **(H2).** *As residents with dementia have a higher need for support in terms of managing everyday life and social contacts, the staff-to-resident ratio for registered nurses is even more decisive for them than for residents not suffering from dementia*.

## 2. Materials and Methods

### 2.1. Data and Study Sample

This study used secondary data that were collected in the cooperative web-based learning project inQS (indikatorengestützte Qualitätsförderung). This was a project of the Diocesan Caritas Association in Cologne (Germany) and the Institute for Knowledge-Based Systems and Knowledge Management at the University of Siegen (Germany) [47]. This project aimed for indicator-based quality promotion using indicators that have been legally required nationwide since 1 October 2019 to assess the quality of care [27].

Selected data collected in winter 2019 were available for the present analyses. Our sample included 30 nursing homes from the federal state of North Rhine-Westphalia, with 2519 residents in total. The indicator-based quality assessment of facilities was based on a full survey, i.e., the inclusion of all residents; nevertheless, 220 residents were excluded from assessment, because they were in the deceasing phase, lived in the corresponding facility less than 14 days, were in hospital for more than 21 days, or were in short-term care. Specified caregivers in the facilities collected fully completed survey forms from the remaining 2299 residents. Using statistical plausibility checks [48], as well as local audits [49], the quality and traceability of the collected data were evaluated. An external expert group additionally controlled and approved the data, and 517 of the 2299 survey forms were excluded, since we decided to only study the highly vulnerable but over-represented group of residents aged over 80 years of age.

**Figure 1:**
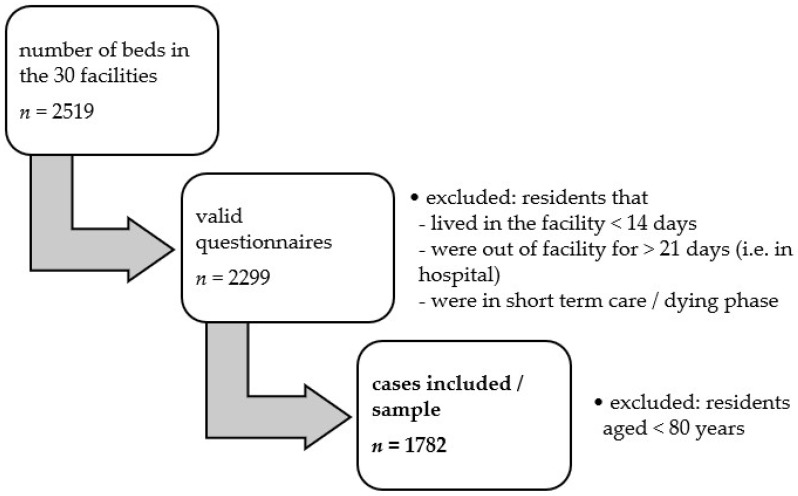
Sample extraction.

In addition, structural data were collected from the long-term facilities. The final dataset for the present study thus included data from 30 nursing homes and 1782 residents (Figure 1). The project coordinators strictly pseudonymized the data. All facilities provided consent to use their data in aggregated form. All respondents provided their written consent to data collection and analysis.

### 2.2. Model Specification

The staffing situation in a facility can reflect the case-mix of its residents [40]. The amount of care may also be distributed differently, for example, to residents who have the most need or to residents who have the most chance of maintaining their abilities [40]. Since facility variance can affect patient care and since residents are nested within nursing home facilities, a multilevel analysis was performed that includes both individual and facility-specific factors. This type of analysis was conducted in order to analyze which characteristics determine the abilities of the residents. Multi-level analyses assume that residents within the same nursing home facility experience similar settings. Staff-to-resident relationships should therefore be examined simultaneously at the resident level and facility level [40]. The staffing level of a facility can be considered as a contextual variable that influences but does not dictate the level of care provided to individual residents. We thus evaluated the effect of the professional staff-mix available in the facility and the amount of care provided to each resident by means of nurse staffing levels.

On the residents’ level, the analysis required the operationalization of seven constructs: sex, age, degree of care, diagnosis of dementia, listlessness due to depressive mood, independent movement in the living area, as well as the abilities to independently maintain everyday life and social contacts. Degrees of care are anchored in German law and assessed in five degrees, from 1 = slight impairment of independence to 5 = most severe impairment of independence with special requirements for nursing care. The dichotomous variable on dementia asked about the presence or absence of this cognitive impairment. Listlessness due to depressive mood was a variable used in the inQS-project and measured on a four-point scale, from 0 = never to 4 = daily. Listlessness was included as a control, since it can be assumed that listlessness due to depressive mood leads to less interest in independently maintaining everyday life and social contacts. Listlessness in a depressed mood is shown, for example, by the fact that a person has hardly any interest in the environment, hardly shows any initiative, and needs motivation from others to do something. This does not include that people with purely cognitive impairments, e.g., dementia, need impulses to start or continue an action. The same holds true for independent movement in the living area, which was rated on a four-point scale from 0 = independently to 3 = dependently. Without the ability to independently move around the living area, maintaining social contacts and everyday life becomes more difficult. A minimum of eight meters was defined as a reference value for normal walking distances within the living area.

Concerning the maintenance of everyday life and social contacts, residents in this study were rated on a four-point scale from 1 = independently to 4 = dependently as to whether they can consciously organize their daily routine according to individual habits and preferences, and if necessary, adapt to external changes (1), assure a day and night rhythm (2), keep themselves occupied (3), make plans (4), interact with people in direct contact (5), and interact with people outside the immediate environment (6). Cronbach’s alpha for this scale was 0.94. We therefore decided to calculate a sum score also ranging from 1 to 4. This sum score formed the dependent variable in our analyses.

Regarding the facilities, we accounted for the number of beds as well as the provision of specialized living units. The latter play a role concerning the care of residents with dementia [24,50]. Specialized living units are divided into integrated, segregated, and the offer of both concepts. In integrated concepts, residents with and without dementia are cared for together. In contrast, in segregated care concepts, residents with dementia are cared for separately from other care recipients [24].

In accordance with Backhaus et al. [23], on the staff side, the total nurse staffing levels and the professional staff-mix were considered. The qualification levels of the staff contained: registered nurses (RN), nursing assistants (NA), and additional care staff (ACS). RN in Germany either have an academic background or have completed a three-year training with a state final examination in one of the two following nursing professions: Health and medical nurse according to the Nursing Act/Geriatric nurse according to the Geriatric Care Act. NA are absolving a one-year training. The ACS in the 30 included Caritas’ facilities run a supervised two-week internship. The latter group is employed to support the other staff and to activate and assist older people in their daily living. ACS do not carry out any medical or nursing activities. Nurse staffing levels comprise the ratio of nursing staff to residents, with each level of qualification (RN, NA, and ACS) being analyzed separately [23]. Since pure head count is of little value, in this study, we chose to calculate full-time equivalents per patient for each of the three levels of qualification. Professional staff-mix is defined as the ratio of RN to the total number of nurse staff [23], with RN, NA, and ACS all measured in full-time equivalents [3].

### 2.3. Statistical Analysis

We conducted the statistical analysis with Stata 16.0 (2019) [51]. The first step included a descriptive analysis of the variables concerning the residents and facilities in the sample. In Table 1 and Table 2, categorical variables are shown as percentages and continuous variables as means with standard deviations. The second step comprised a Spearman’s rho correlation analysis. The results of the latter allowed us to (a) identify initial trends, (b) prove consistencies with the research literature, and (c) create a basis for the multi-level regression analysis.

Thirdly, we conducted a multi-level analysis for the calculated sum score (dependent variable). Here, data were assigned to two levels—resident level and facility level—and we calculated different models. Independent variables consisted of staff-to-resident ratio, professional staff-mix, as well as resident-level covariates and facility-level covariates. The latter comprised degree of care, listlessness, independent movement in the living area, diagnosis of dementia, total number of staff, and provision of specialized living units.

After calculating a null random-intercept-only model (I) (without predictors), we determined the intraclass correlation (ICC) to specify the proportion of variance in the sum score that is due to the facility-level groups (level 2). Afterwards, we successively computed the association between individual-level (model II) and sum score, as well as facility-level (model III) and sum score, respectively adding the variables (degree of care, dementia, listlessness, independent movement for model II, as well as dementia concept, total number of staff, staff-to-resident ratio for each level of qualification, and professional staff-mix for model III) step by step. In the combined model (IV—combining the variables from level 1 and level 2), we added the variables successively. Since our second hypothesis stated that the staff-to-resident ratio for RN is even more decisive for residents with dementia than for residents not suffering from dementia, the factor dementia was put on a random slope. Setting a level-1 variable to random slope allows the effects to vary across the facilities. Hence, the explanatory variable in the random slope model can have a different effect for each group. Finally, we conducted the analysis separately for residents with dementia and residents without dementia (models IV.a and IV.b).

The interclass correlation index (ICC), the Likelihood-ratio test (Lr test), as well as the total variance and variance at levels 1 and 2 were examined for every model. Variables were tested concerning multicollinearity, homoscedasticity of the level 1 residuals, and symmetry of the total residuals. Statistical significance level was set at α = 0.05 for all calculations.

## 3. Results

Data of 1782 people older than 80 years and living in a long-term care facility in North Rhine-Westphalia (Germany) were included. Table 1 descriptively shows participants’ characteristics. The study sample consisted of 88.46% (*n* = 1416) women and 20.34% (*n* = 366) men. Residents were aged 88.14 years on average (±5.03).

**Figure 2:**
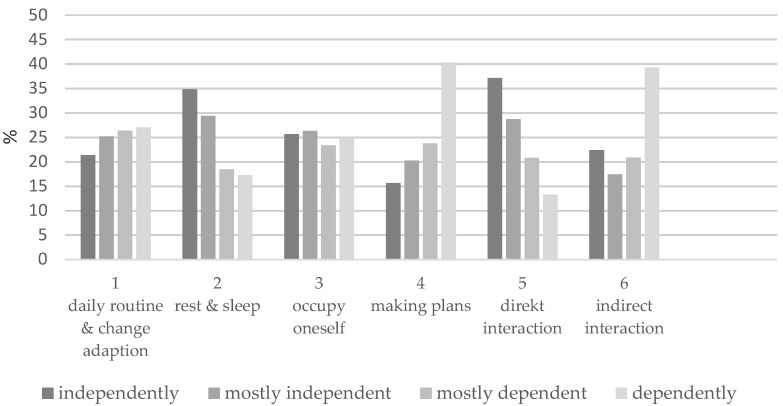
Residents’ abilities to organize everyday life and maintain social contacts.

Figure 2 shows the results for the six items of the dependent variable (the formed sum score). It becomes apparent that residents differ greatly in four items: in items 4 (making plans) and 6 (indirect interaction), most residents show little or no independence in their abilities (65% and 60%). In items 2 (rest and sleep) and 5 (direct interaction), on the other hand, we observed more independence (64% and 62%). Items 1 (daily routine and change adoption) and 3 (occupy oneself) are relatively balanced.

The descriptive results for the facilities and staff are shown in Table 2. The size of the nursing homes ranged from 20 to 184 beds, with 55.43% of the nursing homes providing 76–100 beds. In total, the 30 nursing homes employed 891 caregivers. Facilities altogether employed very few NA compared to RN and ACS.

Besides sex and age that were not significantly correlated, the variables at the resident level showed a moderate correlation with the sum score. Dementia was the only variable negatively associated with the sum score. Three variables on the facility or staff side showed a significant positive but very low correlation with the sum score: ratio RN (r = 0.119, *p* < 0.001), staff-mix (r = 0.08, *p* < 0.001), and total number of staff (r = 0.077, *p* < 0.001).

A multi-level regression analysis was performed to predict residents’ ability to maintain everyday life and social contacts based on individual and facility characteristics. This calculation intended to test for the two hypotheses and to answer our central research question of to what extent nursing staff levels influence residents’ maintenance of everyday life and social contacts.

Table 3 shows the results of the multiple regression analysis. Before adding independent variables, the null model (I) indicated a significant variation in the sum score across facilities (χ^2^ = 109.43, *p* < 0.001). The ICC of 0.10 indicated that 10% of the variance in the sum score results from the grouping variable (facility). With only resident-level variables included, model II showed statistical significance for all the variables (χ^2^ = 54.51, *p* < 0.001). The ICC decreased to 0.05. As expected from the correlation calculation, dementia was negatively associated with the sum score. Since the sample was homogeneous (>80 and mostly female), we assumed that the variables sex and age have little influence. In calculations not shown here, the Lr test for model II indicated that it is irrelevant whether we include or exclude these two variables. The predictive power of the model II (with four level-1 predictors) was about 60% greater at level 1 than in the null model (I).

Model III and onwards included the level-2 variables (facility and staff variables). Neither the number of beds nor the inclusion of a specialized care unit in the facility played a role. Since we observed multicollinearity in terms of VIF for staff-mix and total number of staff, we decided to exclude these variables from the model (see Appendix A, Table A1). Afterwards, the VIF for the included variables ranged between 1.30 (ratio NA) and 1.08 (ratio ACS). The ratio of RN was the only significant variable in this model regardless of whether the other ratios were included in the regression or not. The ICC of model III (χ^2^ = 79.66, *p* < 0.001) ranged at 0.07, and thus decreased compared to the null model (I). Model II was not nested in model III, indicating that model II is superior to model III. The predictive power of the comparison model (with five level-2 predictors) was about 30% greater at level-2 than in the null model.

The combined model IV put dementia on a random slope. All variables at the residential level remained significant. In addition, the category “segregated” with regards to the specialized care unit became significant. Since it was not relevant for the analysis, the variable number of beds was excluded from the analysis here. The ICC of model IV (χ^2^ = 53.69, *p* < 0.001) ranged at 0.13, and was thus higher than in the null model (I). The Lr test indicated that both model II and model III were nested in the combined model (IV). The proportional reduction in the prediction error due to the covariates was 6%.

In a last step, we ran a model separately for residents with (IV.a) and without (IV.b) dementia (Table 3). The results revealed that having a diagnosis of dementia plays a role concerning the ratio of RN and its impact on the sum score. While for the group of residents with dementia (χ^2^ = 7.40, *p* < 0.003), the ratio of RN was not significant, it was at the 10% level (*p* = 0.052) for the group of residents not suffering from this disease (χ^2^ = 32.03, *p* < 0.001). The concept of segregated care for dementia residents remained significant for the group of residents with dementia (*p* = 0.024). The ICC of model IV.a was 0.03, and the ICC of model IV.b was 0.07. The residual variance as well as the R^2^ were comparable for model IV.a (0.35 and 0.63) and model IV.b (0.37 and 0.61).

In summary, all models showed significant effects of the level-1 variables on the sum score. Positive effects were found for degree of care (β = 0.34–0.36; *p* < 0.001), as well as listlessness (β = 0.04–0.12; *p* < 0.05) and independent movement (β = 0.23–0.28; *p* < 0.001). The effect of dementia was strongest in all resident variables and did not differ between model II and model IV (β = −0.56; *p* < 0.001). Dementia was the only effect in the models that turned out to be negative. 

For the level-2 variables, only the staff-to-resident ratio for RN and the presence of a segregated unit varied in terms of significance. All other variables consistently showed no significant effects on the sum score. The effect regarding segregated units was almost equal between models IV, IV.a, and IV.b (β = 0.21–0.22; *p* < 0.05). The effect for staff-to-resident ratio RN, however, differed considerably across the models in which this variable became significant: β = 3.56, *p* < 0.01 for model III (level-2 variables only), and β = 1.61, *p* < 0.1 in model IV.b.

**Figure A1:**
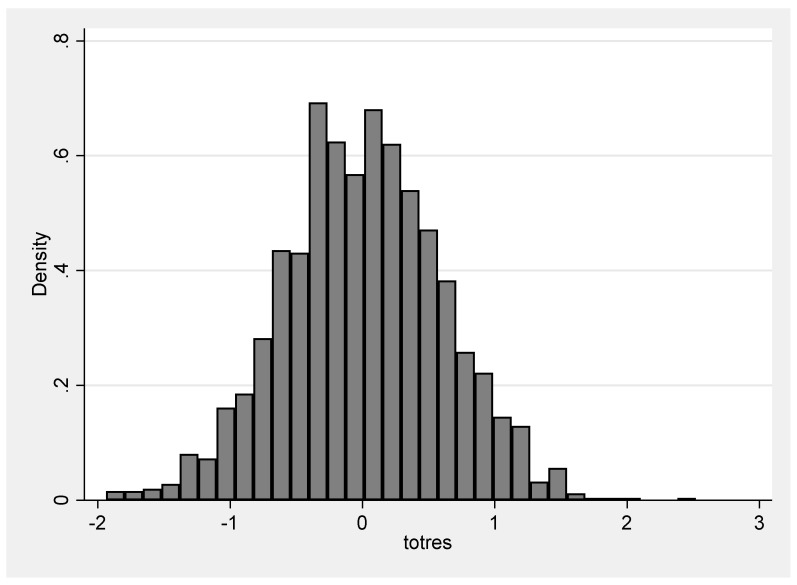
Distribution of total residuals.

**Figure A2:**
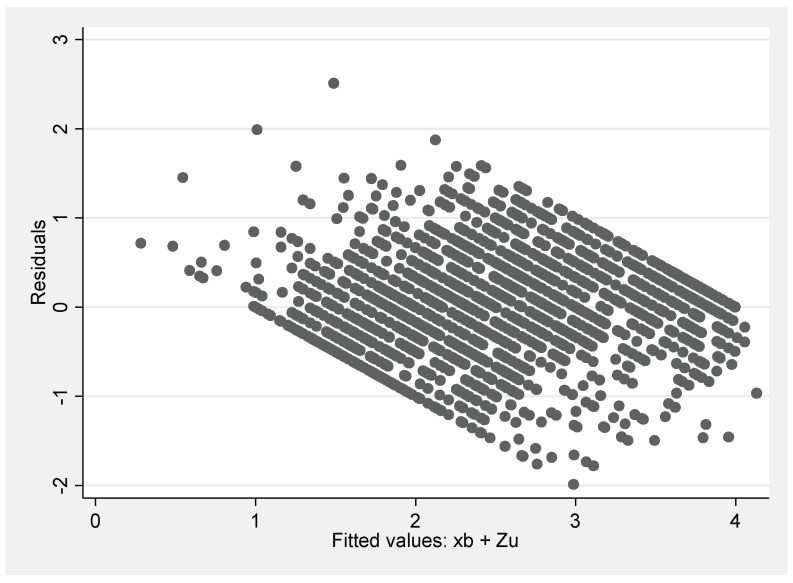
Homoscedasticity.

Model diagnostics were performed and visualized. Multicollinearity in terms of VIF was prevented during the analysis (see Appendix A, Table A1), the error terms were symmetric (see Appendix A, Figure A1), and homoscedasticity (see Appendix A, Figure A2) could be excluded.

## 4. Discussion

The present analysis showed that the staff-to-resident ratio for RN was significantly associated with residents’ ability to organize everyday life independently and to maintain social contacts while living in a nursing home for residents aged over 80 years and without dementia. None of the other staff-to-resident ratios influenced the results concerning residents with or without dementia. The staff-to-resident ratio for RN furthermore did not differ in significance for residents without dementia, regardless of whether we included or excluded the staff-to-resident ratio for NA or ACS. Claiming that the strongest relationships exist between RN and residential outcomes, our findings are in line with those of Konetzka et al. [15], as well as Harrington et al. [22]. We could thus confirm our first hypothesis.

The fact that the staff-to-resident ratio for RN did not play a role concerning residents with dementia may be due to the fact that it is often difficult to interpret their wishes and needs, especially when it comes to social aspects. It could be assumed that residents with dementia are already so restricted in their everyday and social abilities that, independently of the staff, they can no longer maintain the examined abilities. Furthermore, RN often are not very involved in the direct care of residents [7,17,23] and residents with dementia do in fact need a lot of direct care. Direct care is often performed by NA, who, however, were hardly represented in our sample and for which the national shortage is most pronounced [34]. For residents without dementia, on the other hand, RN can actively promote independence.

This implies that the data did not confirm our second hypothesis. Contrary to our assumption, the staff-to-resident ratio does not play a more decisive role for residents with dementia. Rather, what resulted as significant here was whether or not the facility provided segregated care units, or in other words, cared for dementia residents separately from other residents. However, this result has to be interpreted with caution since the group of facilities that offered only segregated care was very small in this study (5 facilities with a total of 181 residents), while 10 facilities (with 614 residents in total) offered both concepts. We could, however, not determine how many residents were cared for in a segregated or an integrated setting in this case. The majority of the residents (*n* = 987) lived in a facility that offers integrated care units only. Interestingly, the effect for the segregated unit differed little between residents with dementia (IV.a with 0.21, *p* = 0.024) and residents without dementia (IV.b with 0.22, *p* = 0.089), but was more significant for the group of residents with dementia.

The number of studies investigating the impact of specialized care units is very small for Germany. A very recent study, however, pointed out that such home layouts have an influence on how easy or difficult it is for residents to socially interact [8]. Zimmermann and Kelleter [24] showed for nursing home facilities in North Rhine Westphalia that in segregated concepts, there are fewer falls, a lower proportion of the use of belt restraints, and a lower rate of pressure ulcers compared to traditional concepts. A previous German comparative study by Weyerer et al. [52] also identified advantages for maintaining the activity level of dementia patients.

It is important to note that studies focusing on specialized care units use different approaches in investigating staff ratios. We observed that the staff-to-resident ratio for RN in the present dataset was particularly high in facilities with an exclusively segregated concept (4/5 facilities ranked in the top 40% of the ratio of RN, 3/5 in the top 15%). Given that the concept of segregated care units is likely to provide more patient-centered care, our results presumably are in line with those of Zimmermann and Kelleter [24], Zimmermann et al. [3], and Weyerer et al. [52]. In conclusion, this type of patient-centered concept is more likely to enable dementia patients to maintain everyday life and social contacts than an integrated one.

As Zimmermann et al. [3] found for falls, we found that differences between residents with dementia and without dementia and residents living in different care units exist with regard to the influence of RN. However, our results with regard to dementia were the opposite to those of Zimmermann et al. This suggests that body-related skills are more likely to be maintained through a high staff-to-resident ratio concerning RN than social and planning skills. According to Kjøs and Havig [53], the increased workload associated with residents with dementia can lead to an “increased focus on daily care and medical treatment”. This increased focus can then negatively impact the provision of activities. Health limitations such as dementia are difficult to reverse in old age and most often imply a continuous process of deterioration in the residents’ general condition, even if numerous caregivers are available at the facility [40].

In this study, the explained level-2 variance was not crucial. Our results illustrate that individual abilities have a much greater influence on how well older people succeed in organizing their everyday lives independently and maintaining their social contacts even while living in a nursing home. In all the models calculated, the variables taken into account at the residential level were significant with regard to the sum score. In addition, the predictive power for the comparator model was about 60% greater than for the null model at level 1, and about 30% greater at level 2. Dementia (models II and IV), as well as degree of care (models IV.a and IV.b), accounted for the largest influence on level 1. Listlessness was the least important factor. Model IV best explained the relationship between residents’ abilities and staffing characteristics (R^2^ = 0.60). Further splitting the model into subgroups showed that the staff-to-resident ratio was more important for residents without dementia (R^2^ = 0.61), and offering a segregated unit was more important for residents with dementia (R^2^ = 0.63). In their qualitative study on the barriers to maintain social contacts in nursing homes, Abbott et al. [31] pointed out that it is not only the staff that plays a role. Personal interests, daily and long-term resources, health status, and the desire for certain activities or contacts are also decisive. In addition, according to the theory of socioemotional selectivity, the interest in social contacts changes over time in older people, with less search for new and more search for emotionally fulfilling contacts [31]. By controlling for listlessness due to depressive symptoms, as well as the ability to independently move in the living area, we were able to control for two variables potentially influencing our dependent variable on an individual level. The database of this study did not allow for an investigation of personal preferences and interests, which would certainly be beneficial to include in future studies.

In their review on the influence of staffing levels on quality of life outcomes, Backhaus et al. [23] stated that the cross-sectional design of many studies may be responsible for the few effects identified on the facility site. This may also be true for the present study. It is therefore strongly recommended to conduct similar studies longitudinally. In addition, Backhaus et al. [23] concluded that quality of care outcomes may differ concerning nursing sensitivity. At first glance, pressure ulcers, falls, and unintentional weight loss, for example, must clearly be more sensitive in terms of the influence of staff than the abilities of residents analyzed in this study. In the era of COVID-19, however, staff play an increasingly significant role for residents’ everyday life and social contacts. Thus, examining the influence of staff has become even more important than before the pandemic. It should be emphasized that a comparison of studies conducted before and after the outbreak of the COVID-19 pandemic is relevant to disentangle the effects of the pandemic on the organization of everyday life and the maintenance of social contacts. While the indicator of maintaining everyday life and activities is also used in other countries (e.g., in the American reporting system “Nursing Home Compare”, which delivers information on and results of quality audits of nursing homes on the internet for users to compare), the subject of social contacts is not used in this form as a quality indicator for nursing outcome quality in other countries. It is, however, taken up in resident surveys to record quality of life and well-being, especially concerning autonomy and social contacts [12]. It should be considered here that in pandemic times, an expansion of classic quality indicators in nursing homes might be appropriate.

Castle and Engbert [41] stated that staff and residents have a closer bond in smaller facilities than in large facilities. This implies that smaller nursing homes are more capable of meeting residents’ individual needs. Our data did not confirm this fact since the number of beds in the facilities was not linked to any of the other variables. After moving into a nursing home, however, residents’ activity levels often decrease. Although staff may not be the sole determining factor here, they can encourage residents to remain as active as possible. Through biographical work, staff can identify residents’ everyday habits and favored social contacts. As Zirves and Pfaff [54] showed, the positive affect of residents can be positively influenced when residents participate and are involved in various activities. However, it is important that they get the chance to freely decide whether they want to participate or not, and thus control their everyday life and social contacts [8].

Workforce planning in healthcare is a top priority issue in international policy organizations. Nursing, taken as a whole, is the health profession that provides the most direct care [55]. Since effective nursing strategies are expected to improve the performance of healthcare organizations, the RN4CAST study used simulations to look at the relationship between hospital nurses and patients, as well as between the skill level of these nurses, their work environment (e.g., job satisfaction, burnout), and patient outcome [55]. Such an approach would also be advisable for care in nursing homes, since in view of the staff shortage in this area, it must also be considered how the working conditions affect the staff and, via this, the resident outcomes [36]. In the course of their study, Rothgang et al. [28] found that many tasks in nursing homes are not carried out, are not carried out completely, or are carried out under time pressure. They therefore developed an algorithm which, depending on the number of residents, indicates how many nursing staff a facility needs to be able to provide a professional level of care. This algorithm is intended to replace the staffing ratios valid in Germany with a needs-based staffing mix. Such personnel assessment procedures will probably be highly relevant in the future, not only in Germany. However, there is one important aspect they cannot measure: the actual competence of nursing staff as well as the continuity and coordination of competence in nursing home facilities. For this reason, it is indispensable to also focus on the development of competence measurement procedures [21,23,40] and of interventions to improve the working environment of nursing care staff (e.g., being involved in decision-making processes, promote skilled interdisciplinary communication, enhancing coping strategies, self-awareness, and emotional intelligence), as summarized by Barrientos-Trigo et al. [36].

Finally, the results of the present study can be incorporated into organizational processes and the planning of personnel capacities. Several implications can be derived for practice: First of all, a more detailed differentiation with a more detailed assignment of tasks is needed in order to define nursing staff qualification levels. Three levels of qualification (as used in the present study) as a measure are not sufficient. It thus would be necessary to adapt the existing qualification profiles in the facilities or their classification according to the risks for care. The existing staff capacities should also be multi-professionally oriented so that risks and resources of residents can be recognized and, if necessary, compensated for or redirected in the sense of the SOC. This should be accompanied by a more targeted development of staff competences with regard to the independent organization of everyday life and the maintenance of social contacts of the residents. 

### Limitations and Strengths

The interactions between residents and caregivers are complex and manifold. Consequently, it is difficult to disentangle whether the number of caregivers assigned to each resident determines the residents’ outcome, or whether the residents’ outcome determines the number of caregivers assigned to the resident [40]. Furthermore, due to their age, the residents analyzed are all highly vulnerable. Thus, facilities in this study sample may be homogeneous in terms of resident structure but heterogeneous in terms of staffing. The cross-sectional design of our study prevents an analysis of cause-and-effect relations and is subject to the risk of endogeneity, a fact that various authors have already pointed out [23,25,40]. Longitudinal studies in this field of research are highly recommended to overcome potential bias.

In agreement with Rothgang et al. [34], we considered the most important structural factor (quality and quantity of staff) according to Donabedian’s model. However, since we analyzed secondary and hard structural data, additional variables were not included. This study only examined the available resources of staff and not the overall context of the organization, which can, for example, reflect the individual competencies, expertise, and morale of the staff, the extent to which the institution is managed in a well-rounded manner, and, for example, uses the latest technologies for communication and participation in social life. Characteristics such as staff turnover, the proportion of agency staff, the distribution of working time among the residents, and the various activities that arise, were not considered either even though other studies clearly highlight the importance of these aspects [21,39,41]. As Aiken et al. [35] emphasize, staffing in hospitals should be considered as a dimension of the work environment, i.e., a broader organizational aspect, which could be a starting point for future research on the topic discussed here. Another limitation was that all facilities had participated in inQS voluntarily and were affiliated with the Caritas Association, a catholic welfare organization [3]. This implies a possible sampling bias.

Zimmermann et al. [3] indicated that regional disparities in care outcomes between federal states in Germany exist. Thus, it may be difficult to transfer the results of this study to other federal states or countries. Even though nursing homes diverge worldwide, for example in terms of organizational structure, staffing, and resident case-mix [14], the results are important beyond the German and European context because of three strengths: The analyses provided significant results for a very specific group of individuals as well as a specific topic, for each of which little research has been conducted up to date. Our results consequently contribute to a better understanding of everyday and social skills of the oldest population. Moreover, multilevel models are optimal for separating facility characteristics from individual resident characteristics. Finally, the dataset provided a unique sample with very good data quality. Our results provide an approximation of the impact of different caregivers on the maintenance of the examined residential abilities.

## 5. Conclusions

The present explorative study provides new data on a specific group of residents aged over 80 years and living in long-term care, as well as on staff levels and staff qualification in the facilities these residents live in. While the impact of staff on residential outcomes has been analyzed in many studies, current research lacks data of how staff-to-resident ratios influence residents’ ability to maintain their everyday life and social contacts. We conducted a preliminary yet innovative study which indicated that for residents without dementia, the ratio of RN, and for residents with dementia, segregated care, were the strongest predictors for residents to maintain their everyday life and their social contacts.

The newly developed instrument by Rothgang et al. is intended to replace the current staffing ratio of 50% of RN in German nursing homes with individual skill mixes in the facilities. If the facility’s staff-mix is aligned with the residents’ individual case mix, the successful implementation of the frequently demanded resident-centered care becomes viable. This, in turn, may also improve residents’ control over their individual organization of everyday routines and the maintenance of social contacts.

## Figures and Tables

**Table 1:** Descriptive characteristics at the residents’ level.

Variables	% (*n*)	Mean	SD	Min	Max
**Age (in years)**	100 (1782)	88.14	±5.03	80	110
**Age (in years for sex)**					
**Women**	79.46 (1416)	88.46	±0.136	80	110
**Men**	20.34 (366)	86.90	±0.235	80	99
Degree of care *	100 (1782)	3.47	±1.03	1	5
0	0.90 (16)				
1	0.34 (6)				
2	16.44 (293)				
3	33.00 (588)				
4	31.93 (569)				
5	17.40 (310				
**Dementia Diagnosis**	53.25 (949)				
**Listlessness**	100 (1782)	1.71	±1.04	1	4
never	62.91 (1121)				
once a week	13.24 (236)				
several times/week	14.14 (252)				
daily	9.71 (173)				
**Independent movement**	100 (1782)	2.18	±1.20	1	4
independently	41.36 (737)				
mostly independently	22.28 (397)				
mostly dependently	12.96 (231)				
dependently	23.40 (417)				
**Sum score**	100 (1782)	2.50	±0.98	1	4

* Degree of care: 0 = none, 1 = slight impairment of independence, 2 = considerable impairment within independence, 3 = severely impaired in independence, 4 = most severe impairment of independence, 5 = most severe impairment of independence with special requirements for nursing care.

**Table 2:** Descriptive characteristics at the facility level.

Variables	% (*n*)	Mean	SD	Min.	Max.
**Number of beds**	100 (1782)	97.30	±34.62	20	184
**Dementia care**					
integrated	55.39 (987)				
segregated	10.16 (181)				
both	34.46 (614)				
**TNS**		52.68	±17.71	13	99
RN		27.40	±10.55	6	61
NA		3.45	±2.64	0	11
ACS		21.40	±9.66	1	39
**Ratio staff-to-resident**		0.55	±0.13	0.38	1.11
ratio RN		0.23	±0.04	0.125	0.38
ratio NA		0.03	±0.02	0	0.07
ratio ACS		0.15	±0.06	0.03	0.43
**Professional staff-mix**		0.42	±0.08	0.22	0.77

TNS = total number of staff, RN = registered nurses, NA = nursing assistants, ACS = additional care staff.

**Table 3:** Regression results.

Model	Null Model (I)		Model II (Restricted) ****		Model III		Model IV (Random Slope for Dementia)		Model IV.a (Residents with Dementia)		Model IV.b (Residents without Dementia)	
Variables	Est. [95% CI]	*p*	Est. [95% CI]	*p*	Est. [95% CI]	*p*	Est. [9% CI]	*p*	Est. [95% CI]	*p*	Est. [95% CI]	*p*
dementia			−0.56 [−0.62–0.50]	0.000 ***			−0.56 [−0.65–0.47]	0.000 ***				
degree of care			0.35 [0.32–0.39]	0.000 ***			0.35 [0.32–0.39]	0.000 ***	0.36 [0.31–0.41]	0.000 ***	0.34 [0.29–0.39]	0.000 ***
listlessness			0.08 [0.05–0.11]	0.000 ***			0.07 [0.05–0.10]	0.000 ***	0.04 [0.00–0.07]	0.049 *	0.12 [0.08–0.17]	0.000 ***
independent movement			0.25 [0.22–0.28]	0.000 ***			0.25 [0.22–0.28]	0.000 ***	0.23 [0.19–0.27]	0.000 ***	0.28 [0.24–0.33]	0.000 ***
dementia care												
segregated					0.18 [−0.19–0.54]	0.340	0.21 [0.05–0.37]	0.009 **	0.21 [0.03–0.39]	0.024 *	0.22 [−0.03–0.48]	0.089
both					0.11 [−0.13–0.36]	0.366	0.08 [−0.03–0.18]	0.157	0.08 [−0.03–0.20]	0.159	0.04 [−0.13–0.21]	0.649
staff-to-resident ratio RN					3.56 [1.27–5.84]	0.002 **	0.67 [−0.04–1.76]	0.227	0.36 [−0.91–1.62]	0.582	1.61 [−0.01–3.23]	0.052
staff-to-resident ratio NA					−1.52 [−7.64–4.61]	0.627	1.66 [−1.08–4.41]	0.235	1.85 [−1.24–4.94]	0.240	1.44 [−2.88–5.78]	0.512
staff-to-resident ratio ACS					0.39 [−1.16–1.94]	0.619	0.24 [−0.50–0.98]	0.522	0.45 [−0.38–1.29]	0.285	−0.44 [−1.64–0.75]	0.466
Test Statistics												
total variance/residual variance	0.97		0.55		0.94		0.38		0.35		0.37	
level-1 R^2^			0.60									
level-2 R^2^					0.30							
total R^2^			0.43		0.03		0.60		0.63		0.61	
ICC	0.10 [0.56–0.17]		0.05 [0.03–0.1]		0.07 [0.04–0.13]		0.13 [0.04–0.31]		0.03 [0.00–0.07]		0.07 [0.04–0.15]	
χ^2^	109.43	0.000 ***	54.51	0.000 ***	79.66	0.000 ***	53.69	0.000 ***	7.40	0.003 **	32.03	0.000 ***

* *p* < 0.05, ** *p* < 0.01, *** *p* < 0.001; **** age and sex were excluded. Est. = estimates, RN = registered nurses, NA = nursing assistants, ACS = additional care staff, R² = coefficient of determination (R squared), χ^2^ = chi-square, ICC = intraclass correlation coefficient.

## Data Availability

For data protection reasons, we are tied to a confidentiality agreement. The raw data used thus cannot be made publicly available. The collection, use, and analysis of the data has been described in detail by Kelleter [47], Kelleter and Herfen [49], as well as Kelleter, Zirves, and Zenkert [27].

## Appendix A

**Table A1:** Multicollinearity.

Variable	VIF	1/VIF		Variable	VIF	1/VIF
Staffmix	20.20	0.05				
TNS	17.52	0.06				
Number of beds	14.53	0.07		Number of beds	1.10	0.09
Ratio ACS	13.37	0.07		Ratio ACS	1.08	0.92
Ratio RN	11.90	0.08		Ratio RN	1.10	0.09
Ratio NA	4.26	0.23		Ratio NA	1.30	0.77
Dementia care concept	2.22	0.45		Dementia care concept	1.09	0.91
Mean VIF	12			Mean VIF	1.14	
